# Efficacy and Reliability of Retrograde Intrarenal Surgery in Treatment of Pediatric Kidney Stones

**DOI:** 10.7759/cureus.3719

**Published:** 2018-12-11

**Authors:** Musa Ekici, Berat Cem Ozgur, Aykut Bugra Senturk, Cemil Aydin, Arzu Akdaglı ekici, Muhammet Yaytokgil, Mehmet M Baykam

**Affiliations:** 1 Urology, Hitit University, Erol Olcok Training and Research Hospital, Corum, TUR; 2 Urology, Health Sciences University, Ankara Training and Research Hospital, Ankara, TUR; 3 Anesthesiology, Hitit University, Erol Olcok Training and Research Hospital, Corum, TUR

**Keywords:** kidney stone, retrograde intrarenal surgery, pediatric kidney stone

## Abstract

Introduction

Surgical treatment of pediatric kidney stones has dramatically changed in recent years due to the miniaturization of surgical instruments and the availability of intracorporeal lithotriptors. Retrograde intrarenal surgery (RIRS) technique is now considered an effective and minimally invasive procedure in renal stones. However, in the pediatric age group, the number of studies on this subject is very limited. The aim of this study was to evaluate the efficacy and safety of the RIRS in the treatment of kidney stones in children.

Material and methods

The data of 25 pediatric stone patients who underwent RIRS with the diagnosis of kidney stones were analyzed retrospectively. Demographic characteristics, operative data, and success rates of the patients were recorded.

Results

Fourteen (56%) of the cases were male and 11 (46%) were female. The mean age was 10.43 ± 4.26 (3-15) in boys and 10.18 ± 4.92 (4-16) in girls. Eleven stones (46%) were in the left kidney and 14 (56%) in the right kidney. The mean stone size was 10.08 ± 4.33 mm (4-23). Stone localizations were renal pelvis in 15 (60%) cases, upper calyx in four (16%) cases, middle calyx in five (20%) cases, and lower calyx in one (4%) case. The mean operation time was 41.20 ± 6.96 minutes (30-60), the mean duration of scope was 17.40 ± 3.85 seconds (10-30), and the mean hospital stay was 2.32 ± 0.63 days (2-4). Three patients (12%) had undergone percutaneous nephrolithotomy (PCNL), and two (8%) patients underwent shockwave lithotripsy (ESWL) before this surgery. In six (24%) patients, a double J (DJ) catheter was inserted due to stenosis at the lower end of the ureter. Body mass index (BMI) of these patients was below 18. An access sheath was implanted in six (24%) patients in the second operation. In 18 cases, the first operation was performed with a direct flexible renoscope. In all cases, a postoperative DJ catheter was inserted. Postoperative fever was observed in one (4%) patient, and ureteric steinstrasse was observed in one (4%) patient. The stone-free rate was achieved as 17% (68%) after the first operation and 100% after the second RIRS session.

Conclusion

RIRS appears to be an effective and reliable method in the pediatric age group. However, there is a need for multicentre studies involving more cases.

## Introduction

Ureteroscopy was first performed by Enrique Perez Castro in 1980, and the first retrograde intrarenal surgery (RIRS) operation was performed by Huffman et al. in 1983 [1]. Important advancements were provided in RIRS with the development of holmium laser in 1995 [2]. The main goal in the treatment of renal calculi is to provide maximum stone-free rate with minimal morbidity. RIRS has become an important treatment option for kidney stones in pediatric patients with the development of new-generation ureteroscopy and holmium laser. It is an effective method in the proximal ureter, collecting duct system, and, especially, lower calyx calculi. The risk for complications is significantly lower with RIRS and the complications are mostly minor [3]. RIRS is a less invasive intervention to access renal calculi compared with percutaneous nephrolithotomy (PNL) and open pyelolithotomy [4]. In this study, we aimed to demonstrate the efficacy and reliability of the RIRS operations performed in our hospital in pediatric patients.

## Materials and methods

The data of pediatric patients who underwent RIRS at the Hitit University Corum Erol Olcok Training and Research hospital between 2015 and 2018 were retrospectively evaluated and analyzed. The necessary consents were received preoperatively from parents of the patients, and in addition to routine investigations, urinalysis and urine culture were ordered in all patients scheduled for operation. Intravenous urography (IVU) was performed in all patients to evaluate the anatomy of the collecting system. Stone size was calculated as the longest measurement calculated at a single dimension on ultrasonography or direct urinary system graphy (DUSG). The operation was performed under general anesthesia in all patients. The patients were placed in the lithotomy or frog-leg position. First, diagnostic ureterorenoscopy (URS) was performed in all patients with a 6.4 Fr ureterorenoscope. Ureteropelvic junction (UPJ) was preferably accessed where it could be done using a guidewire (0.038 hydrophilic-coated glidewire), and a retrograde pyelogram was taken. In the case where ureteral orifice could not be passed through, a double J (DJ) catheter was inserted for passive dilatation, and the operation was terminated and postponed for two weeks. In the cases where the ureter was accessed without problem, it was advanced up to the UPJ and the wire was left into the pelvis. Then the same process was repeated; a second guidewire was inserted into the pelvis renalis to provide passive dilatation in the ureter. A 7.5 Fr Flex 2 (Karl Storz GmbH& Co. KG, Tuttlingen, Germany) flexible ureterorenoscope was then slid over the guidewire and advanced up to the pelvis through the scope and endoscopic visual control. When the pelvis was reached, the guidewire was withdrawn and diagnostic endoscopy was performed. The stones were fragmented using a 273-micron fiber with the help of laser (Sphinx Holmium Laser 30w, Lisa Laser Products, Germany). The fragmentation was continued until the largest stone diameter was dropped to 2 mm, and the fragments were left to fall spontaneously in order to minimize the complications related to repetitive in and out movements. An access sheath (9.5 Fr Flexor® Ureteral Access Sheath, Cook Medical) was used in the surgery performed after two weeks in patients who underwent passive dilatation by inserting the DJ catheter in the first operation. In these cases, the fragmented stones were removed through the sheath utilizing a basket. At the end of the operation, a DJ catheter or a mono J catheter by cutting one tip was inserted into the patients according to their age and height.

Operational time was recorded as the time from scrubbing and positioning of the patients followed by entering through the urethra with the ureterorenoscope to the insertion of the DJ catheter. The scope duration was calculated as the recording of the total imaging during the operation in seconds.

Statistical analysis

Statistical analysis of the data obtained from the study was performed with SPSS (Version 22.0, SPSS Inc., IL, USA, Licence Hitit University). Descriptive statistics were expressed as the mean ± standard deviation for the continuous variables or median (min-max) and the number and percentage for the categorical variables depending on the distribution of the data. Normality was evaluated with Shapiro-Wilk test. Since parametric test hypotheses were not provided for the comparison of two independent mean values, non-parametric Mann-Whitney U test was used. Whether body mass index (BMI) could be used to predict if the ureter would be accessed in the first session was statistically investigated with the receiver operating characteristic (ROC) analysis method. The area under the curve (AUC) and 95% confidence intervals of this area were calculated with the ROC analysis method. The significance of the variables in the determination of the risk group was taken as AUC >0.500 in the analysis (0.9-1: excellent, 0.8-0.9: good, 0.7-0.8: moderate, 0.6-0.7: weak, and 0.5-0.6: failure). The sensitivity and specificity of these variables were calculated for the success of the classification of the risk group. Youden index (maximum sensitivity and specificity) was used to determine the best cut-off point in the ROC analysis. *p* < 0.05 values were considered statistically significant.

## Results

Twenty patients (80%) had an indication for primary RIRS. Three (12%) patients previously underwent PCNL, and they had residual stones. Two patients previously underwent ESWL, but the stones could not be broken. All stones were in various localization in the kidneys, and the ureteral calculi were excluded from the study. Since access could not be achieved in six (24%) cases in the first session, a DJ catheter was inserted and these patients were operated in the second session. Because postoperative stone street occurred in one (4%) patient, URS was performed in the second session. Stone-free status was achieved in 17 (68%) patients in the first session and in all patients in the second session. The mean scope time was 17.40 ± 3.85 (10-30) seconds. One patient (4%) developed stone street and one (4%) patient fever. The mean duration of hospitalization was 2.32 ± 0.63 (2-4) days. The mean operational time was 41.20 ± 6.96 (30-60) minutes. Table 1 lists the characteristics of the stones and also the operation findings.

**Table 1:** Characteristics of the stones and the operation findings

	Groups	*n *(%)	Mean ± SS (min-max)
Age	Boy	14 (%56)	10.43 ± 4.26 (3-15)
	Girl	11 (%44)	10.18 ± 4.92 (4-16)
Stone side	Right	11 (%44)	
	Left	14 (%56)	
Stone localization	Renal pelvis	15 (%60)	
	Upper-pole calyx	4 (%16)	
	Middle calyx	5 (%20)	
	Lower calyx	1 (%4)	
Stone-free rate	1. session	18 (%72)	
	2. session	25 (%100)	
Postoperative complication	Fever	1 (%4)	
	Stone street	1 (%4)	
Mean stone size (mm)			10.08 ± 4.33 (4-23)
Mean operational time (minutes)			41.20 ± 6.96 (30-60)
Scope time (seconds)			17.40 ± 3.85 (10-30)
Mean duration of hospitalization (days)			2.32 ± 0.63 (2-4)

Comparison of the BMI levels between the groups created according to the success of the access to the ureter in the first session is given in Table 2. The mean BMI value was found as 18.38 ± 0.48 (18-19) in patients in whom ureters could be accessed in the first session, and this value was significantly lower than the mean value of 21.36 ± 2.18 (17-25) in patients in whom the ureters could be accessed in the second session (*p *= 0.003, Table 2).

**Table 2:** Comparison of BMI values between the groups created according to success of the access to the ureter in the first session BMI: body mass index

Access in the first session	*N*	Mean ± SD	Median (Min-Max)	P value
No	19	21.36 ± 2.18	21.50 (17-25)	0.003*
Yes	6	18.38 ± 0.48	18.20 (18-19)	

Since there was a significant difference between the mean values, the cut-off value was investigated with the ROC analysis. Results of the ROC analysis are given in Table 3. According to the results of the ROC analysis, the best cut-off value was found as 19.35 with Youden index. Sensitivity was found to be 100% and specificity 84.2% for a cut-off value of 19.35. The rate of success of access to the ureter in the first session was found to be 100% when the BMI value was higher than 19.35. The rate of failure of access to the ureter in the first session was found to be 84.2% when the BMI value was lower than 19.35.

**Table 3:** Results of ROC analysis ROC: receiver operating characteristic

Area under the curve	Standard error	P value	95% Confidence interval	
			Lower limit	Upper limit
0.890	0.071	0.005	0.752	1.000

## Discussion

In recent times, open surgery for renal calculi has been replaced by less invasive interventions such as ESWL, PNL, and RIRS in both adults and children [5-7]. Although ESWL has been accepted as the first-line treatment in stones less than 20 mm in the 1980s, it has several disadvantages compared to the RIRS such as the negative effects on renal parenchyma and adjacent organs, anesthesia administration in places out of the operating room, and the need for more than one ESWL. In addition, increased stone burden and stone stiffness decrease the success chance of ESWL [5,8-9]. In our study, two (8%) patients resistant to ESWL underwent RIRS and complete stone-free rates were obtained. Therefore, we think that RIRS is a good treatment option for the stones resistant to ESWL.

Percutaneous nephrolithotomy (PNL) has been one of the most commonly preferred treatment options, especially in the treatment of renal calculi greater than 2 cm since Woodside et al. published their first series in 1985 [10]. Initially, urologists have approached to PNL treatment in the pediatric age group unwillingly, because the use of large instruments in kidneys of small sizes in children may cause parenchymal damage, leading to renal dysfunction, and there are several risks with this method including radiation exposure and severe complications. However, Jackman et al. underlined that creating a smaller tract will cause less tissue and nephron injury, and this was more important especially in pediatric patients with fragile kidneys of small sizes and described mini-perc technique [11]. It has been shown in many pediatric PNL series performed later that the use of small-size instruments decreased the risk of intraoperative complication, and even new modifications have been described such as tubeless PNL and mini-perc to further decrease the complication rates [12-14]. Today the open treatment has been replaced by PNL in pediatric patients for high stone burden in all age groups. However, despite all these modifications and high success rates, major complications such as adjacent organ injury, severe bleeding, and urosepsis have been reported up to 10% during PNL, and whether this method is actually a minimal invasive is still controversial [15]. Therefore, RIRS is an alternative treatment option to PCNL. In addition, this is also an effective treatment method for residual stones after PCNL. In our study, RIRS was performed for the residual stones from PCNL in three patients, and stone-free status was obtained in these patients.

Ureteroscopic stone treatments have been introduced in adults at the end of the 1970s, although the first results of the use of this technique in children have been published by Ritchey et al. in 1988 [16]. Although this method has been first used only in the treatment of ureteral calculi, it has been introduced in the treatment of renal calculi with advancements in the flexible endoscopic technology. The results of the treatment of renal and ureteral stones have been given together in the first series, although series about the use of flexible URS (f-URS) only in pediatric patients have been published in recent years [17]. In our study, we also included the patients who were treated only for renal calculi.

The use of ureteral access sheath facilitates the replacement of ureteroscope processes in adults, both increasing the stone-free rates and shortening the operational time [18]. In addition, it has been proposed that this method provides a continuous flow, decreasing intrarenal pressure and related complications. Unsal et al. provided stone-free status in three patients using a ureteral access sheath and observed no complication related to the sheath [19]. However, in a study by Traxer et al. with adult patients, ureteral damage was found in 167 of 369 patients using an access sheath. The authors reported that this damage was seen by seven folds especially in patients who were not stented before the procedure [20]. Wang et al. showed that the use of the access sheath increased intraoperative complications in 96 patients with a mean age of 13. Particularly, extravasation and perforation were more common in patients in whom an access sheath was used [21]. Although there is a risk for the development of stricture with the use of the sheath, no stricture development has been reported in pediatric patients. In our series, we used a ureteral access sheath in six patients and removed the fragments. In these patients, ureter could not be accessed in the first surgery, and passive dilatation was made by inserting the DJ catheter. We did not use a ureteral access sheath in 19 patients, reduced the size of the stones, and left them to pass spontaneously. Long-term results of the leaving fragments after RIRS are still debatable [22-23]. It has been claimed that especially the fragments left in the lower calyx – particularly in the presence of cystine stones – may cause stone formation again [24]. In our study, we left the fragments to spontaneously drop in 19 patients. At follow-up, the stone street was observed in one patient due to the fragments, and the stones were removed from the lower end of the ureter in the second session. The development of the stone street was attributed to the high stone burden of the patient (23 mm).

Several mechanisms play a role in the etiology of fever observed despite all the measures taken after the urinary system stone surgery. Among these, anesthesia-related complications may be seen after all types of surgeries, and fever due to the release of endotoxins following fragmentation is seen especially after surgeries such as PNL, URS, and f-URS [25]. Different theories have been proposed for the explanation of fever especially due to f-URS. The most important of them is the high collecting system pressure during the operation. This problem is more common in the cases where access sheath is not used [26]. Herein, in our clinic, we use high-pressure irrigation with a pump as a measure in patients without the access sheath inserted and aspire the irrigation fluid with flexible renoscopy through the kidney using a 20-cc injector at certain intervals. In our study, resolving of fever seen in one patient with conservative approaches and no growth in blood and urine cultures suggested that the non-infectious factors were prominent.

Dilatation of the ureteral orifice is another problem in pediatric patients. Although no case has been reported in the literature, it has been theoretically proposed that active dilatation of the orifice and even the procedure itself may cause reflux [27]. In our study, we preferred passive dilatation using a DJ stent instead of active dilatation in six patients in whom the ureter could not be accessed. These six patients who received double J stents were operated again after two weeks and we had not any difficulty during inserting access. Unsal et al. applied active dilatation in five patients, one of them developed perforation, but they found no stricture or reflux in their patients during long-term follow-up [19].

Failure to access the ureter and reach to stone with f-URS is also reported as a complication [27]. In our clinic, we access the ureter first by a semi-rigid ureterorenoscope and then access the kidney by seeing via the guide with a flexible device. In the case of failure, we insert a ureteral double J stent for passive dilatation and postpone the operation. We think that the prediction of whether the ureter could be accessed in the first surgery will both protect the patients against the complications and shorten anesthesia duration. It has been reported in the literature that BMI has no effect on success or complications after RIRS [28-29]. However, BMI has not been evaluated for prediction of whether the ureter would be accessed. We evaluated whether BMI could be used in prediction access to the ureter. The rate of success of access to the ureter in the first session was found as 100% when the BMI was higher than 19.35. The rate of failure of access to the ureter in the first session was found as 84.2% when the BMI was lower than 19.35. We believe that as the number of our patients was small, if this clinical result is supported with a larger case series, BMI may provide insights about whether access to the ureter could be provided.

The limitation of this study is the small number of patients. Another limitation is the lack of a control group or ESWL and/or PNL groups for comparison. Further prospective, randomized controlled studies are needed for f-URS to become more popular. Finally, multicenter studies will increase evidence grade.

## Conclusions

The RIRS method that has become more popular in the recent years with the advancements in technology is increasingly placed at first rank in the preference of urologists as an efficient and reliable method and an alternative option to ESWL in small renal calculi in pediatric patients and to PNL in large renal calculi. When making decisions for the treatment method, the size, composition, and the localization of the stone, anatomy of the urinary system, patient preference, existing equipment, and surgeon’s experience must be taken into account.
